# Mutation order in acute myeloid leukemia identifies uncommon patterns of evolution and illuminates phenotypic heterogeneity

**DOI:** 10.21203/rs.3.rs-3516536/v1

**Published:** 2023-11-06

**Authors:** Matthew Schwede, Katharina Jahn, Jack Kuipers, Linde A. Miles, Robert L. Bowman, Troy Robinson, Ken Furudate, Hidetaka Uryu, Tomoyuki Tanaka, Yuya Sasaki, Asiri Ediriwickrema, Brooks Benard, Andrew J. Gentles, Ross Levine, Niko Beerenwinkel, Koichi Takahashi, Ravindra Majeti

**Affiliations:** 1. Department of Medicine, Division of Hematology, Stanford University, Stanford, CA, USA; 2. Department of Biomedical Data Science, Stanford University, School of Medicine, Stanford, California, USA; 3. Biomedical Data Science, Institute for Computer Science, Free University of Berlin, Berlin, Germany; 4. Department of Biosystems Science and Engineering, ETH Zurich, Basel, Switzerland; 5. SIB Swiss Institute of Bioinformatics, Lausanne, Switzerland; 6. Division of Experimental Hematology, Cincinnati Children’s Hospital Medical Center, Cincinnati, OH, USA; 7. Department of Pediatrics, University of Cincinnati, Cincinnati OH USA; 8. Department of Cancer Biology, Perelman School of Medicine, University of Pennsylvania, Philadelphia, PA, USA; 9. Human Oncology and Pathogenesis Program, Molecular Cancer Medicine Service, Memorial Sloan Kettering Cancer Center, New York, New York, USA; 10. Louis V. Gerstner Jr. Graduate School of Biomedical Sciences, Memorial Sloan Kettering Cancer Center, New York, New York, USA; 11. Department of Leukemia, The University of Texas MD Anderson Cancer Center, Houston, TX.; 12. Cancer Institute, Stanford University School of Medicine, Stanford, CA.; 13. Institute for Stem Cell Biology and Regenerative Medicine, Stanford University School of Medicine, Stanford, CA.; 14. Department of Pathology, Stanford University, Stanford, CA, USA; 15. Department of Medicine, Stanford Center for Biomedical Informatics Research, Stanford University, Stanford, CA, USA; 16. Center for Hematologic Malignancies, Memorial Sloan Kettering Cancer Center, New York, New York, USA; 17. Leukemia Service, Department of Medicine, Memorial Sloan Kettering Cancer Center, New York, New York, USA

## Abstract

Acute myeloid leukemia (AML) has a poor prognosis and a heterogeneous mutation landscape. Although common mutations are well-studied, little research has characterized how the sequence of mutations relates to clinical features. Using published, single-cell DNA sequencing data from three institutions, we compared clonal evolution patterns in AML to patient characteristics, disease phenotype, and outcomes. Mutation trees, which represent the order of select mutations, were created for 207 patients from targeted panel sequencing data using 1 639 162 cells, 823 mutations, and 275 samples. In 224 distinct orderings of mutated genes, mutations related to DNA methylation typically preceded those related to cell signaling, but signaling-first cases did occur, and had higher peripheral cell counts, increased signaling mutation homozygosity, and younger patient age. Serial sample analysis suggested that *NPM1* and DNA methylation mutations provide an advantage to signaling mutations in AML. Interestingly, *WT1* mutation evolution shared features with signaling mutations, such as *WT1*-early being proliferative and occurring in younger individuals, trends that remained in multivariable regression. Some mutation orderings had a worse prognosis, but this was mediated by unfavorable mutations, not mutation order. These findings add a dimension to the mutation landscape of AML, identifying uncommon patterns of leukemogenesis and shedding light on heterogenous phenotypes.

## Introduction

Acute myeloid leukemia (AML) has a dismal prognosis, with a five-year overall survival of approximately 30% (1). The poor outcomes are in part due to AML being a heterogeneous disease, with substantial variability between cases and in the subclones of an individual case (2–4). Recent studies have elucidated the clinical consequences of individual mutations in AML (4) and their interactions (5), but little research has evaluated whether modes of leukemogenesis like mutation order, rather than presence of mutations, are associated with clinical features and outcomes (6,7). Preleukemic cells often harbor mutations related to epigenetic modification, which usually occur before those related to cell signaling (8,9), but whether mutations can also occur in atypical orders, such as signaling mutations first, and the relationship between mutation order and phenotype in AML are poorly characterized.

In a related group of disorders, myeloproliferative neoplasms (MPNs), variable mutation order is relevant to disease phenotype and provides insight into pathogenesis. The order of mutations in *TET2* and *JAK2* is associated with *JAK2* homozygosity, patient age, and cell proliferation (6). The composition of hematopoietic stem and progenitor cells (HSPCs) also differs, with single-mutant cells dominating HSPCs in *TET2*-first cases but not in *JAK2*-first cases, suggesting that *TET2* mutations offer a fitness advantage in HSPCs compared to *JAK2* mutations (6).

Here, we analyzed AML samples for similar patterns related to mutation order by aggregating large single-cell DNA sequencing (scDNAseq) datasets and using computational tools to create evolutionary trees. We characterize the co-occurrence and order of select mutations and the relationship between mutation order and several clinical features.

## Materials and Methods

### Data

Previously published scDNAseq data of patients with AML came from the MD Anderson Cancer Center (123 patients, 154 samples) (10), Stanford University (14 patients, 38 samples) (11), and Memorial Sloan Kettering (MSK) Cancer Center (91 patients, 116 samples) (12) (Supplementary Figure 1). Three more Stanford patients were included because this analysis included secondary AML, and each contributed three samples (diagnosis, remission, relapse). The sequencing has been described in detail in each respective study. Briefly, data were generated using Mission Bio’s Tapestri platform, and FASTQ files had been processed using Mission Bio’s Tapestri Pipeline v1. Zygosity was determined using the GATK HaplotypeCaller (13) and did not distinguish homozygosity from loss of heterozygosity.

All samples from MSK were processed using a custom targeted 31-gene sequencing panel, and 64 samples from MD Anderson were processed using a custom 37-gene panel. All other samples underwent sequencing using a 19-gene AML-specific panel created by Mission Bio (Supplementary Table 1). All panels included those 19 genes, and for the initial descriptive analyses, all data and mutations were considered.

### Identifying driver mutations

Variants were included if both 1% of cells were mutated (11), and the lower bound of a confidence interval for the number of cells containing the mutation was greater than 10 (12). Variants were considered driver mutations using prior criteria (14,15) (Supplementary Methods), or if they had experimental evidence supporting their pathogenicity (Supplementary Figure 2). Variants were excluded if they are not associated with AML but either appeared in most patients in a dataset or were repeatedly mutated in a low percentage of cells (Supplementary Methods, Supplementary Figure 3).

### Modeling mutation acquisition

Single Cell Inference of Tumor Evolution (SCITE) (16,17) was used to create a mutation tree for each patient. Mutation events were assumed to occur at most once and not to revert to wildtype during a patient’s course (infinite sites assumption). We assumed that zygosity has minimal impact on mutation calling and on mutation order inference, so zygosity was ignored when creating trees. When multiple samples were available for a patient, samples were merged into a single mutation matrix where mutations absent at one timepoint but not another were assumed wildtype. When variants of unknown significance were available, they were included in the mutation matrix to inform tree architecture but not for downstream mutation order inference. See Supplementary Methods for additional modeling details.

### Modeling FLT3-ITD variants

Several samples had multiple distinct insertion sequences in *FLT3* exons 14 or 15, where *FLT3* internal tandem duplication (FLT3-ITD) mutations occur. However, we suspected that different ITDs in the same patient often represented the same ITD event for the purposes of evolutionary analyses. This is because seemingly distinct ITDs insertions usually shared similar DNA sequences, and different datasets had substantially different numbers of ITDs per patient, suggesting batch effects (Supplementary Figure 4). Thus, we merged ITDs from a patient if insertions started at the same locus and were subsequences of another insertion, if they were all terminal events from the same parent event in a tree, or if not merging ITDs resulted in more poorly supported connections in the tree (“Tree Analysis” below, Supplementary Methods, Supplementary Figure 4).

### Tree analysis

Driver mutations, which were summarized as either the genes or biological pathways affected (Supplementary Table 2), were analyzed as trees with R v4.3.0 using the igraph package (18). When merging graphs, the size of an edge or vertex reflected the number of times the same sequence of events starting from the root node was observed in the entire dataset. A mutation was considered “early” if no single-cell mutations preceded it (Supplementary Methods). The binomial test and exact multinomial test (19) were used to evaluate doublet and triplet mutation orders, respectively. When analyzing the percentage of cells with a certain mutation, the denominator was the number of cells with a call for that mutation. Comparisons between mutation order and clinical characteristics were tested with a Wilcoxon rank-sum test unless otherwise specified.

To ensure that the data supported the paths between every driver mutation in the same clone, the percent of cells with a later mutation that contained the earlier mutation, the “cell support,” was calculated for each mutation pair. Paths with <50% cell support were “low-support.” Variants in low-support paths were excluded based on how many low-support paths they contributed to and their distal position in the tree (Supplementary Methods, Supplementary Table 3, Supplementary Figure 5).

## Results

### Creating mutation trees

Three targeted single-cell AML DNA sequencing datasets were merged (10–12), resulting in 207 patients with AML who had at least two driver mutations, 275 samples, 823 mutation events, and 1 639 162 cells (Supplementary Figure 1). Most samples were cytogenetically normal (53%), and datasets had similar patient demographics but varied in distributions of laboratory values, treatment, and sample availability at diagnosis (Table 1). Although all sequencing panels covered 19 commonly mutated genes, datasets differed in the size of the sequencing panels (Supplementary Table 1), number of cells per sample, and the Tapestri Pipeline allele dropout estimate (Supplementary Figure 6). After aggregating these datasets, mutations were represented in similar proportions as in the TCGA (20) and BeatAML (21) studies, except for enrichment in mutations common in AML, such as in *NPM1* and *FLT3*, and in low-level signaling mutations (Supplementary Figure 7A); for instance, 58% of *KRAS* mutations were in <10% of the corresponding sample’s cells.

**Figure 1:**
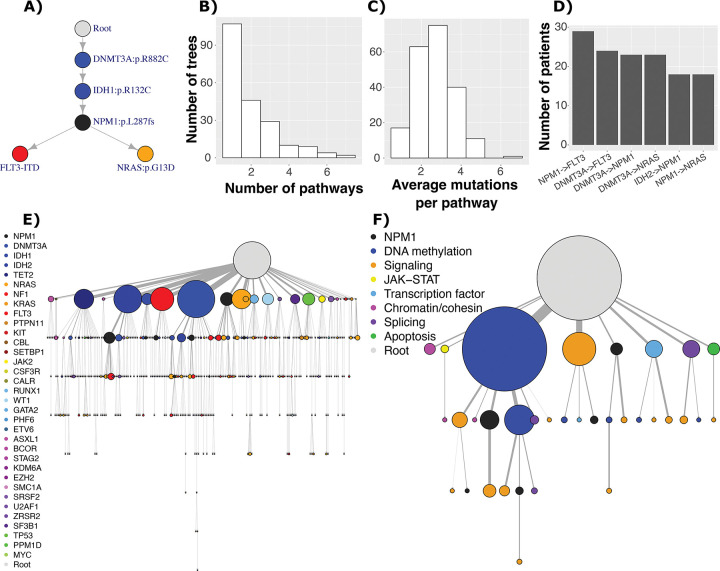
A) Example tree. Distributions of B) the number of distinct evolutionary pathways per tree (number of trees = 207), and C) the average number of mutations per pathway. D) Most common two-gene evolutionary pathways mutated, when mutations were summarized by gene. E) All trees merged, summarized by the gene in which the mutation is present, where size of node represents the number of times a particular pathway occurs, starting from the root node. Colors correspond to mutations, where genes with similar functions have similar colors (e.g. blue shades for DNA methylation and red/orange shades for signaling mutations). F) All trees merged, where the mutation events were summarized by pathway, and only evolutionary pathways with at least five events are depicted.

Using SCITE (16,17), we created a mutation tree from each patient’s mutation matrix (e.g. Figure 1A). The trees had variable numbers of pathways and genes per pathway (Figure 1B–C), and the most common pairwise links between mutations involved *NPM1*, *DNMT3A*, *FLT3*, *NRAS*, and *IDH2* (Figure 1D). These orderings were corroborated by bulk sequencing since differences in VAF (variant allele frequency) from sequencing done using the same samples and variants correlated with differences in the mutated percentage of cells for pairs of variants in the same clones (Pearson correlation 0.57, p = 2 × 10^−51^, Supplementary Figure 7B). Of the 101 trees that had branched evolution, signaling mutations represented 66% of the events that immediately followed a branching point (Supplementary Figure 8A). In contrast, *NPM1* mutations frequently served as a branching point (Supplementary Figure 8B) because *NPM1* often preceded signaling mutations; if branching occurred after an *NPM1* mutation, 93% of such occasions involved a signaling mutation vs. 28% if branching did not occur.

When summarizing mutations to genes (Figure 1E), 224 distinct evolutionary orderings occurred across all patients (e.g. *DNTM3A*→*NPM1* is indistinct from *DNTM3A*→*NPM1*→*FLT3*). Given the complexity of Figure 1E, we merged trees but summarized events according to the biological pathway corresponding to each gene (Figure 1F, Supplementary Table 2). Mutations related to DNA methylation (e.g. *DNMT3A*, *IDH1*/*2*) were frequently early, and terminal events were often signaling mutations. We also noted that DNA methylation mutations often followed other DNA methylation mutations, which was driven by specific types of DNA methylation mutations that are less associated with AML progression (22). For example, while *DNMT3A* R882, *IDH1*, and *IDH2* mutations commonly preceded *NPM1* or signaling mutations (Supplementary Figure 9A), *DNMT3A* non-R882 mutations usually preceded other DNA methylation mutations (Supplementary Figure 9B).

### Pairwise mutation co-occurrence and order

**Figure 2:**
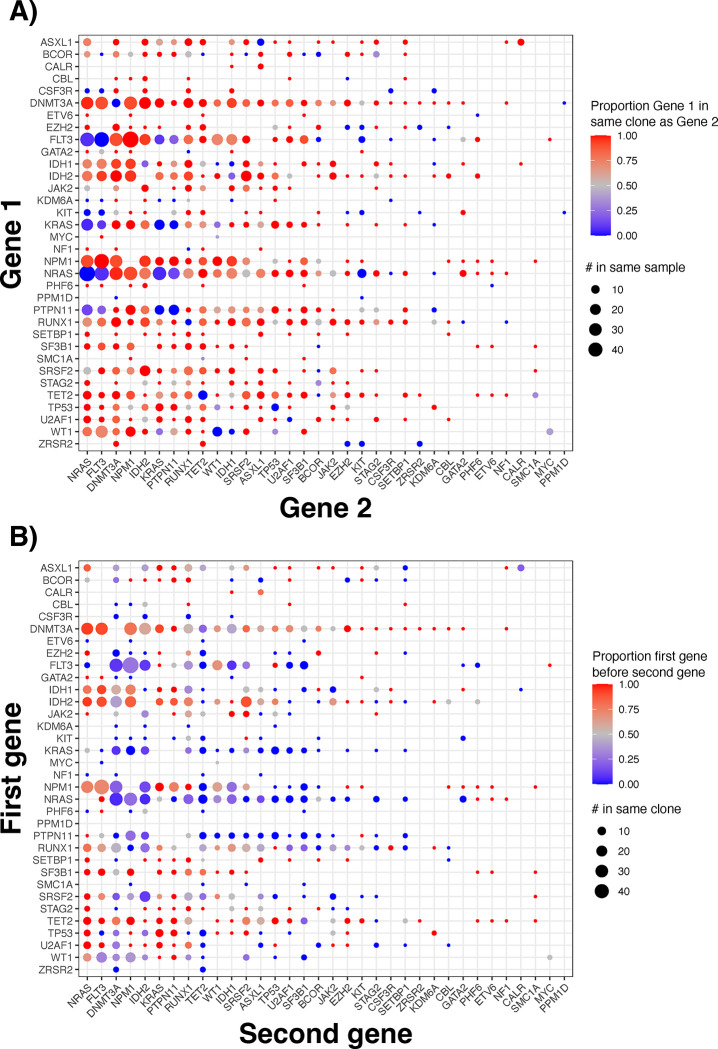
A) Plot showing whether two mutations occur in the same or different clone, summarized by gene. Size of each dot represents the number of times mutations in two genes occur in the same patient sample, and color represents the frequency they are in the same clone. B) Whether one mutation occurs before another mutation. Size of dot represents the number of times they are in the same clone (not just in the same patient sample), and color represents the proportion of times a mutation in a gene on the y-axis came before a mutation in a gene on the x-axis.

To further characterize the co-occurrence of mutations, we analyzed the frequency at which mutations occurred in the same or different clones (Figure 2A). Signaling mutations (Supplementary Table 2) in the same cases typically occurred in different clones. For instance, different *NRAS* mutations occurred in distinct clones in 100% of cases. In contrast, *NPM1* mutations nearly always (>90% cases) co-occurred in the same clone as mutations in signaling genes, DNA methylation genes, or transcription factors (Figure 2A).

Many mutations also often had characteristic orderings relative to each other, such as *DNTM3A* mutations occurring early and signaling mutations occurring late (Figure 2B, Supplementary Table 4), similar to prior work (8). However, transcription factors like *RUNX1* and *WT1* had variable mutation orderings, appearing both before and after mutations that are typically early (e.g. *DNMT3A*) or late (e.g. *FLT3*).

Analyzing the order of mutation trios (rather than pairs) corroborated these findings, where trios often began with DNA methylation mutations and terminated with signaling mutations (Supplementary Table 5). Evolution of *DNMT3A*→*NPM1*→*FLT3* was common, but other mutations trios had variable mutation orderings, like combinations with DNA methylation and splicing mutations.

### Uncommon mutation orders

Although many mutation pairs occurred in characteristic orders, we noted several cases where mutation order deviated from typical patterns, such as when signaling mutations occurred before a DNA methylation or *NPM1* mutation (Supplementary Figure 10).

**Figure 3:**
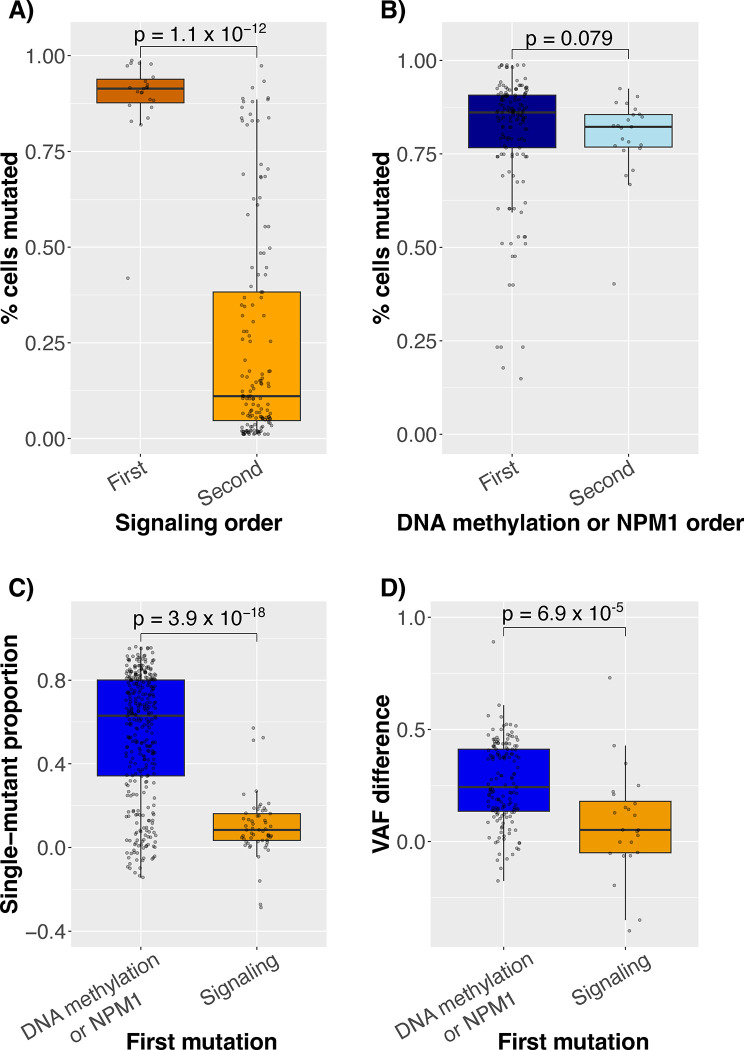
Boxplots showing the A) percentage of mutated cells containing a signaling mutation vs. whether the signaling mutation came before (First) or after (Second) an *NPM1* or DNA methylation mutation. B) Same plot as (A) except that the focus is on the *NPM1* or DNA methylation mutation percent cells mutated. C) Size of a single-mutant clone stratified by which mutation came first. Single-mutant clone size was estimated by subtracting the proportion of cells with each mutation after removing cells where there was no call for the mutation. This plot shows that the single-mutant clones for *NPM1*/DNA methylation-first cases were higher than in signaling-first cases. D) Difference in variant allele frequency (VAF) using bulk sequencing data from the same samples and variants. In A) and B), only diagnostic samples were used since the absolute amount of disease may vary with treatment, and the n = 148 for *NPM1*/DNA methylation-first and n = 23 for signaling-first. In C) and D), since the focus was on relative sizes of clones, all samples were used, with n = 338 and n = 57 for *NPM1*/DNA methylation-first and signaling-first groups, respectively, and because of missing bulk sequencing data in D), n = 151 and n = 24, respectively.

Before characterizing these atypical orderings in detail, we validated their presence. First, if signaling mutations came before *NPM1* or DNA methylation mutations, then the percentage of cells with those mutations should be higher. Indeed, the signaling mutation clone size in diagnostic samples was higher when the mutation came before (vs. after) the *NPM1* or DNA methylation mutations (p = 1.1 × 10^−12^, Figure 3A). Interestingly, the percentage of cells with *NPM1* or DNA methylation mutations was high irrespective of relative signaling mutation order (Figure 3B). Next, if signaling mutations came first, then both the percentage of mutated cells and the bulk VAF should be higher than those of *NPM1* and DNA methylation mutations. Indeed, across all samples and driver mutations, the signaling mutation’s percentage of mutated cells and VAF were higher when it was first (89% [51/57] and 63% [15/24] of pairwise comparisons, respectively) and lower when second (94% [318/338] and 93% [140/151]).

Although these results corroborated the existence of signaling-first cases, the signaling mutation-only clones in the signaling-first cases were consistently small. Using the difference in percentage of mutated cells as a proxy for clone size, the single-mutant clone size was smaller in signaling-first cases than in *NPM1*/DNA methylation-first cases (p = 4 × 10^−18^, Figure 3C). This difference was also corroborated using the difference in bulk VAFs as a proxy for single-mutant clone size (p = 7 × 10^−5^, Figure 3D).

**Figure 4:**
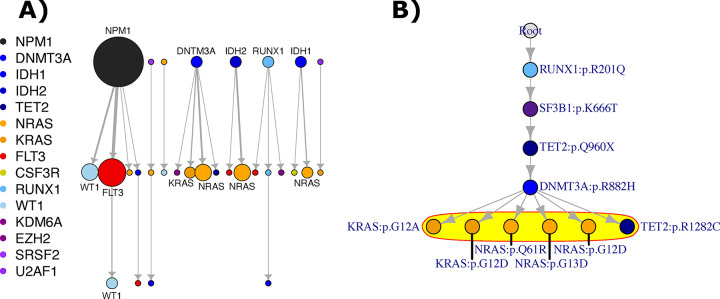
A) All new pathways at relapse across all available paired serial samples in the single-cell dataset (derived from 25 diagnosis/relapse and 15 relapse/relapse pairs, 34 patients total). The top layer of events represents events present in the prior sample, although not necessarily the initial event of a tree, and the lower layers represent events gained on a subsequent sample. Genes with more than one instance are labeled directly. B) Example tree for which serial samples are available, where the events circled in yellow are new events on a subsequent sample.

A similar pattern of single-mutant clone size was previously seen in *JAK2*-first vs. *TET2*-first MPNs, where *JAK2*-first cases had fewer single-mutant HSPCs, suggesting that *TET2* mutation increased the fitness of *JAK2* mutation in HSPCs (6). Thus, we suspected that *NPM1* and DNA methylation mutations offered a selective advantage for signaling mutations among HSPCs in AML. We explored this phenomenon by examining new mutations across serial samples (25 diagnosis/relapse pairs, 15 relapse/relapse pairs, 34 patients, Figure 4A). Most new mutations at relapse were signaling mutations (60%, 21/35), and new signaling mutations tended to arise after a previously present DNA methylation or *NPM1* mutation. When considering all potential nodes in a tree from which signaling mutations could arise (including the possibility of no prior mutations), *NPM1* and DNA methylation mutations disproportionately served as the immediate parent node for a new signaling mutation (9/10 parent nodes, Fisher’s test p = 0.002). For example, in Figure 4B, the *NRAS* mutations arose in the *DNMT3A* clone, despite the *DNMT3A* mutation being present in 41% of the earlier sample’s cells compared to ≥90% of cells for the other mutations. Because signaling mutations disproportionately followed DNA methylation and *NPM1* mutations, *NPM1* and DNA methylation mutations may offer an advantage for signaling mutations in HSPCs.

### Clinical correlates with mutation order

**Figure 5:**
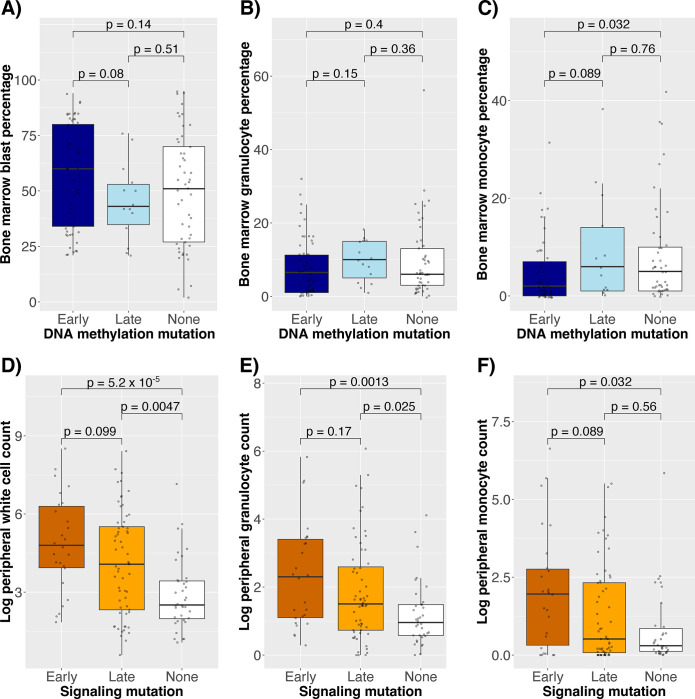
Earlier DNA methylation mutations were associated with higher bone marrow blast percentages while earlier signaling mutations were associated with higher peripheral myeloid cell counts. A-C) Distributions of A) bone marrow blast percentage, B) bone marrow granulocyte percentage, and C) bone marrow monocyte percentage compared to whether a DNA methylation mutation was early, late, or not present in the sample. D-F) Distributions of D) log peripheral blast count, E) log peripheral granulocyte count, and F) log peripheral monocyte count compared to whether a signaling mutation was early, late, or not present.

Because *TET2* mutations change the HSPC balance in MPNs (6), we hypothesized that any advantage conferred by DNA methylation mutations in AML was partially due to expansion of more immature HSPCs, apparent as blasts. To explore this, we compared “late” and “early mutations, which are those that occur with and without any preceding mutations in the scDNAseq data. Indeed, the bone marrow blast percentage was higher in diagnostic samples with early DNA methylation mutations compared to late DNA methylation mutations (p = 0.08, Figure 5A), while the bone marrow granulocyte and monocyte percentages were generally lower (p = 0.15 and p = 0.09, respectively, Figure 5B–C).

In contrast, signaling mutation order (see Supplementary Methods for justification of the “early” and “late” categorization of signaling mutations) was not associated with the bone marrow cell percentages (p ≥ 0.7 for all comparisons), but it was associated with higher peripheral white blood cell (WBC) counts (p = 0.099, Figure 5D). Although peripheral blast counts were higher in signaling-early cases (median 14.8 vs. 3.7, rank-sum p = 0.14), so were the peripheral granulocyte and monocyte counts (p = 0.17 and p = 0.089, respectively, Figure 5E–F). Notably, we consider signaling mutations to be one group for simpler interpretation, but they have different clinical phenotypes, such as early *NRAS*/*KRAS* mutations having higher monocyte counts than later *NRAS*/*KRAS* mutations (p = 0.056), a trend not seen for *FLT3* mutations (p = 0.38).

To ensure that these associations between order and cell composition were not dataset-specific, we used proxies for early and late mutation order, specifically high and low VAFs (cutoff 0.3, previously used to define dominant and clonal mutations (23,24)), for validation in the BeatAML bulk DNA sequencing data (21). Early DNA methylation mutations were indeed associated with higher bone marrow blast percentages (p = 0.00041, Supplementary Figure 11A). In contrast, while early signaling mutations were not associated with bone marrow blast percentage (p = 0.35), they were associated with higher peripheral white blood cells, granulocytes, and monocytes (p < 0.05 for all comparisons, Supplementary Figure 11B-D).

Although these mutation orderings had distinct phenotypes, we also wished to distinguish whether the phenotype was related to the order or the increased clonal burden that resulted from a mutation occurring earlier. Thus, using the scDNAseq data, we performed multiple linear regression adjusting for patient age and the percent of cells with the relevant mutation (Supplementary Table 6). In multivariable analyses, DNA methylation clone size (p = 0.0079), but not mutation order (p = 0.21), was associated with bone marrow blast percentage, suggesting that clone size mediated the association between DNA methylation order and blast percentage (Supplementary Table 6A). In a similar regression, signaling mutation clone size, rather than mutation order, was significantly associated with peripheral blast percentage (p = 0.0084, Supplementary Table 6B). However, signaling mutation order was independently associated with peripheral granulocyte and monocyte counts (p = 0.088 and 0.035, respectively, Supplementary Table 6B), suggesting that the order of signaling mutations, not just the clonal burden, contributed to more mature myeloid cell counts.

**Figure 6:**
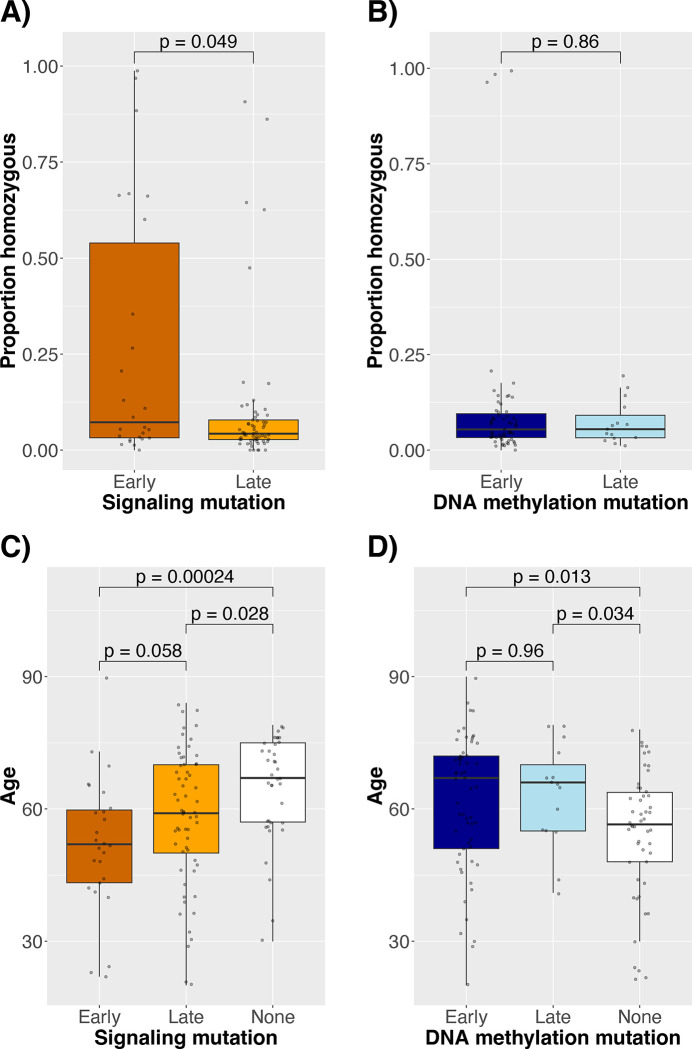
Signaling mutation (A-B) zygosity and patient age (C-D) at diagnosis compared to whether signaling (A, C) and DNA methylation (B, D) mutations were early or late (or there was no mutation, in the age comparison) among diagnostic samples. “Early” means that no mutations are known to occur before it based on the scDNAseq dataset.

We next tested whether mutation orderings in AML could explain other patient and disease characteristics, such as younger age and increasing signaling mutation homozygosity, which are associated with *JAK2*-first MPN cases (6). Indeed, in diagnostic samples with early signaling mutations, signaling mutations were more often homozygous (median 5% vs. 21% of cells homozygous, p = 0.049, Figure 6A), and patients were younger (median 52 vs. 59 years old, p = 0.058, Figure 6C). In contrast, the same patterns did not hold for DNA methylation mutations (Figure 6B,D). Notably, the association with signaling mutation homozygosity was driven by a minority of cases (Figure 6A) and primarily *FLT3* (p = 0.011), for which loss of heterozygosity has previously been associated with poor prognosis (25). Although detecting zygosity in scDNAseq data could be confounded by allele dropout, we found no evidence of this since *FLT3* mutation homozygosity was also not correlated with the number of cells missing mutation calls for the relevant mutation or with sample-level allele dropout (Spearman correlation 0.04 [p = 0.78] and 0.07 [p = 0.65], respectively).

This constellation of evolutionary patterns and clinical correlates involving signaling mutations also creates potential to better understand other mutations. For example, *WT1* mutations contribute to relapse (26) but have an unclear role in AML pathogenesis (27), and we found that *WT1* mutations share many characteristics with signaling mutations. Like mutations in *FLT3* and *NRAS*, *WT1* mutations frequently occurred in *NPM1-*mutant clones (Figure 2A, Figure 4A); early *WT1* mutations often occurred in younger patients; and *WT1*-first cases had small single-mutant clones when co-occurring with *NPM1* mutations (Supplementary Figure 12). In multivariable analyses, early *WT1* mutations were also associated with age and higher neutrophil and monocyte counts (Supplementary Table 6C).

Although we found several phenotype differences associated with mutation order between DNA methylation and signaling mutations, patients with these different orderings did not have significantly different overall survival (Cox regression age-adjusted p = 1 for signaling vs. DNA methylation first). Among relatively prevalent mutation orderings, *SF3B1*→*FLT3* was nearly significantly associated with a worse prognosis after false discovery rate (FDR) correction (age-adjusted hazard ratio 5.6, q-value = 0.056, Supplementary Figure 13A). However, this association was no longer significant after adjusting for the presence of an *SF3B1* mutation (p = 0.44), which itself carries a poor prognosis (4).

Still, exploratory analyses of other phenotypes at diagnosis (Supplementary Figure 13B-E) revealed meaningful associations, such as evolution involving *IDH1/IDH2* mutations and lower granulocyte (median 1.7 vs. 3.0, p = 3.6 × 10^−6^) and monocyte counts (median 1.2 vs. 1.9, p = 0.0063), or orderings with *SRSF2* occurring predominantly in older individuals (median age 73 vs. 59, p = 0.017).

## Discussion

We showed that although AML evolution is heterogeneous, mutations tend to occur in characteristic orders, both at the levels of the genes and the biological pathways involved. This is consistent with prior findings that certain mutations, such as those related to epigenetics, often occur early in evolution whereas signaling mutations occur later (8).

However, we expanded on these findings through analysis of large-scale single-cell sequencing data, identifying important patterns in clonal architecture and how those relate to clinical phenotype in AML. We found that many AML cases are characterized by linear evolution, with branching evolution primarily involving signaling mutations. Our analyses also revealed several cases with atypical or poorly characterized mutation orderings, such as signaling mutations preceding DNA methylation mutations or DNA methylation mutations preceding other DNA methylation mutations. Early signaling mutations were associated with 1) proliferative disease, 2) increased signaling mutation homozygosity, and 3) younger patient age. These results are analogous to previous findings in MPNs (6), but we established these conclusions in a more acute, aggressive, and heterogeneous disease. Additionally, the mutation order framework provided insight into poorly understood mutations, like in *WT1*, which had evolutionary patterns and phenotypic associations similar to signaling mutations but where the associations with age and proliferation were independent of the effects of signaling mutations in multiple regression.

By using serial samples, we also showed that signaling mutations commonly arise in clones containing mutations in *NPM1* and those related to DNA methylation, suggesting that these mutations may offer a relative fitness advantage for signaling mutations in HSPCs. This was further corroborated by the small clone size of single-mutant clones in signaling-first cases. Because the size of the DNA methylation clones correlated with the bone marrow blast percentage in our scDNAseq dataset and the BeatAML dataset, any advantage may be mediated by a shift to immature cells in the bone marrow.

This study has several strengths. First, to our knowledge, this is the largest analysis to date of single-cell DNA sequencing data, an increasingly important data type (28), within a single disease, and the first to benefit from merging multiple clinically relevant datasets together. Second, we leveraged the granular clonal architecture revealed by these data to develop an algorithm to model FLT3-ITD evolution. This is important because the presence of multiple ITDs is associated with a worse prognosis (29), but if multiple ITDs are detected, they may not represent distinct evolutionary events because ITD sequences can be unstable (30) or may be the result of technical artifacts. Third, we used state-of-the-art algorithms to create mutation trees and derive mutation order for each patient’s samples, allowing us not only to identify which mutations tend to occur early vs. late but also to identify the order of mutations in a sample.

Most importantly, this study adds a new dimension to typical analyses of mutations in AML by examining the order of mutations rather than their presence, co-occurrence, or clonal burden, and this order was associated with clinically relevant traits. Although there is tremendous excitement about how patterns of clonal evolution contribute to the disease course (10–12,28,31), it is crucial to distinguish the effects of clonal architecture from the effects of common clinical measurements that can be derived from bulk sequencing. For example, in some analyses, we found that mutation order itself was independently associated with a phenotype, while in others, we found that the presence of clonal burden of select mutations, rather than the mutation order, mediated association with clinical features. Regardless, considering mutation order will likely be clinically useful, especially when selecting targeted therapies. For example, when *IDH* and *FLT3* mutations co-occur, they virtually always occur in the same clone (Figure 2A). Because *IDH* mutations usually come first in evolution (Figure 2B), the cells that have *FLT3* mutations typically also have *IDH* mutations, suggesting that *IDH* could be targeted to treat the *FLT3*-mutant cells. However, if *FLT3* comes first, there could be residual *FLT3*-positive cells if only the *IDH* mutation is targeted.

Our study also has some limitations. First, we focus on mutations in individual genes rather than also analyzing large structural rearrangements, which are important in classifying AML (4). Second, this study does not incorporate single-cell surface protein markers (10,12), which may be helpful to distinguish AML cells from other non-leukemic clonal hematopoiesis cells in a sample (32). However, this limitation would not affect the conclusions of this study since many of the mutations analyzed, such as those in *NPM1*, are specific to AML (33) or are uncharacteristic of clonal hematopoiesis. Third, the available data cannot be leveraged to estimate how quickly the AML evolved, unlike recent whole genome sequencing studies focused on MPNs (34,35). However, by using clinical data, we noted that patients whose disease had early signaling mutations were usually younger, suggesting a faster evolution to AML. Fourth, given the lack of single-cell whole-genome sequencing, we cannot rule out that other driver mutations absent from the sequencing panels that are essential for the clonal evolution were excluded. However, this does not invalidate the orderings and overall trends we observed. Lastly, to identify correlations between mutation order and clinical variables, we used retrospective data, and unknown confounders could explain the observed associations.

Future studies could model AML evolution in the context of surface protein markers (34,35) or gene expression (36), or with either larger targeted sequencing panels or a larger dataset. It also remains unclear how specific treatments, such as targeted therapies, affect the clonal architecture of AML, and this could be studied more closely.

AML is increasingly understood as a heterogenous disease that evolves from other conditions, such as clonal hematopoiesis and myeloproliferative neoplasms. We foresee a future where treatment is decided not only based on what is observed in a case of the disease, but how that disease came to existence. Modeling the development of AML by placing mutations in their context rather than focusing on the traits of a static sample may open new avenues of both clinical and basic research. These large-scale evolutionary models are a step towards that future.

## Figures and Tables

**Table 1:** Characteristics of patients from each dataset either as a proportion of the dataset or as a median and a range.

Variable	MD Anderson (n = 114)	MSK (n = 96)	Stanford (n = 15)

Age	61.5 (21–91)	66.5 (20–86)	53 (22–70)
Female sex	0.39	0.42	0.53
Secondary AML	0.26	0.23	0.07
Stem cell transplant recipient	0.35	0.35	0.53
Diagnosis sample available	0.71	0.36	1
Complex cytogenetics	0.11	0.19	0.07
Abnormal cytogenetics	0.42	0.6	0.21
Treatment at first induction*			
Intensive chemotherapy	0.61	0.46	1
HMA-based therapy	0.38	0.36	0
Other	0.01	0.18	0
Complete blood count*			
WBC count	11.1 (1.1–363)	7.3 (0.5–88.4)	67.5 (1.3–210)
Peripheral blast %	20 (7–94)	20 (0–88)	75 (7–97)
Peripheral monocyte %	8 (0–69)	5 (0–62)	0 (0–9)

* Only diagnostic samples used for these variables.

## Data Availability

Genomic data that were created for this study are available on dbGaP with accession phs002049.v1.p1 and, on Sequence Read Archive with NCBI BioProject ID PRJNA648656. Data from Stanford is being submitted to dbGaP. Clinical data are available on request. Code is on Github at https://github.com/mattschwede/aml-mutation-order.
